# Editorial: Premature Aging and Senescence in Renal Fibrosis

**DOI:** 10.3389/fphar.2021.734892

**Published:** 2021-07-26

**Authors:** Rohan Samarakoon, Paul J. Higgins

**Affiliations:** Department of Regenerative & Cancer Cell Biology, Albany Medical College, Albany, NY, United States

**Keywords:** CKD, SASP, klotho, PAI-1, p53, p21, p16, senolytics

Cellular senescence, defined as the irreversible cell cycle arrest, plays an important role in both development and disease. While acute senescence is important for organogenesis and proper wound healing, perpetuation of the senescent population is associated with extensive transcriptomic reprogramming. The accompanying metabolic changes and acquisition of inflammatory and secretory properties by chronically senescent cells lead to abnormal organ repair, matrix accumulation and tissue fibrosis.

Permanently growth-arrested, phenotypically-altered senescent renal cells are evident in various renal injuries (e.g., ischemic, obstructive, toxin-induced, diabetic and hypertensive) and accumulate in the kidney with increasing age where they are associated with progressive scarring, declining organ function and reduced regenerative capacity. Acute kidney injury (AKI) is a common and frequently life-threatening condition that often progresses to fibrosis and chronic stage disease (CKD). CKD patients, moreover, are more susceptible to AKI as are aging adults and CKD prevalence is significantly higher in older individuals. Although cellular senescence is an important contributor to progressive fibrosis, the underlying mechanisms are not well understood. Furthermore, renal aging and CKD share certain pathophysiologic features suggesting a mechanistic linkage between the two processes. The papers in this Special Issue entitled “Premature Aging and Senescence in Renal Fibrosis” probe the biological basis of this interrelationship. This collection addresses the molecular and pathologic underpinnings of renal aging and the senescent phenotype that contribute to renal injury, failed or maladaptive repair and progressive fibrosis and build on the literature highlighting the role of age-dependent pathology on kidney disease (Kanasaki et al., 2012; Ruiz-Ortega et al., 2020; Mylonas et al., 2021).

The elegant review by Xu et al draws parallels among renal aging, senescence, and CKD progression. The senescence-associated secretory phenotype (SASP), characterized by increased production of inflammatory mediators, matrix modulators and cytokines, orchestrates pathogenic cell-cell communications in the renal parenchyma with predisposition to fibrosis. Targeting the SASP, therefore, is a potentially viable strategy to attenuate fibrosis. Rescue of endogenous inhibitors of senescence such as the anti-aging protein klotho, which is known to be repressed in many nephropathies regardless of etiology, provides another avenue to mitigate fibrosis and the transition to CKD. The recognition that several renal cell types including tubular and endothelial elements as well as interstitial fibroblasts acquire senescent characteristics in aging and disease suggests there may be multiple targets for senotherapy.

Docherty et al draw distinctions between normal and senescent cells in another state-of-the-art review published in this issue. They discuss role of cellular senescence during development as well as in several pathological settings and describe the beneficial impact of acute senescence and the detrimental effects of chronic senescence and SASP in progressive renal disease. Depletion of senescent cells in the kidney, via transgenic as well as pharmacological strategies, attenuates fibrogenesis and improves renal function. Senostatic approaches that target SASP also mitigate CKD progression.

Franzin et al. highlight the involvement of several pathways in premature renal aging including mitochondrial dysfunction, the reno-protective effects of autophagy or “self-eating,” epigenetic events, generation of extracellular vesicles and the DNA damage response. The authors discuss the various pharmaceutical strategies that specifically target and eliminate senescent cells (senolytic therapy) or inhibit expression of paracrine-acting senescence-associated secretory factors (via senostatic/senomorphic drugs). Included is an extensive listing of current senolytic and senomorphic agents; some of which may have utility in the management of CKD and renal transplant patients.

The paper by Maique et al. draws interesting correlations between elevated plasma phosphate and renal senescence. This relationship appears to be mechanistically linked to increases in the CDK inhibitors p21 and p16, upregulation of the profibrotic/senescence-inducing p53 target gene encoding plasminogen activator inhibitor-1 (PAI-1), a member of the serine protease inhibitor gene family (SERPINE1) and a significant decrease in the anti-aging protein Klotho. The data are consistent with the concept that restoration of Klotho levels attenuates development of phosphate-induced renal senescence/fibrosis and, more importantly, may have more widespread applicability as a therapeutic modality for kidney diseases of various etiologies.

Marquez-Exposito et al. address several mechanisms underlying the pathomolecular basis of age-associated increase in AKI which is a significant contributor to mortality in old mice and the >65-years patient population. The authors compared the renal response of young vs. old mice using folic acid-induced AKI as a model system. The extent of Tubular damage, expression of KIM-1 and proinflammatory genes and neutrophil/macrophage infiltration are increased in the AKI kidneys of old mice as was the extent of tubular cell death due to ferroptosis and necroptosis. AKI kidneys displayed evidence of cellular senescence, including increased levels of cyclin dependent kinase inhibitors and p21cip1, expression of specific SASP components (Il-6, Tgf*β*1, Ctgf, and Serpine1) and the DNA damage response marker γH2AX. The authors raise the interesting possibility that molecular senescence in the immune cells (“immunosenescence”) may be a critical element in the increased severity of AKI in old mice. In contrast, expression of renal protective factors klotho and PGC-1α were significantly reduced in elderly AKI mice. These findings indicate that aging is a major factor in the development of a severe form of AKI in response to folic acid and likely due to an increased engagement of proinflammatory-related pathways in older mice and the increased age-associated incidence of senescence cells in the kidney.

Collectively, these papers underscore the recognition that several renal cell types including tubular, glomerular and endothelial elements as well as interstitial fibroblasts acquire senescent characteristics in aging and disease. The diversity of cell types involved in both processes and the various pathways implicated suggests that there may be multiple candidate targets for senescence-based therapeutics. Indeed, senolytic/senostatic therapies are currently in clinical trials with encouraging results. This Special Issue, therefore, is a critical resource which summarizes the pathophysiology, basic underlying mechanisms and potential treatment strategies for progressive aging and senescence-related renal disease.
